# Hypocretin/orexin influences chronic sleep disruption injury in the hippocampus

**DOI:** 10.3389/fnagi.2022.1025402

**Published:** 2022-10-06

**Authors:** Henry Nick, Polina Fenik, Yan Zhu, Sigrid Veasey

**Affiliations:** Department of Medicine and the Chronobiology and Sleep Institute, Perelman School of Medicine, University of Pennsylvania Philadelphia, Philadelphia, PA, United States

**Keywords:** chronic sleep loss, chronic sleep disruption, septohippocampal cholinergic system, amyloid, degeneration

## Abstract

Chronic sleep disruption is a risk factor for Alzheimer’s disease (AD), yet mechanisms by which sleep disturbances might promote or exacerbate AD are not understood. Short-term sleep loss acutely increases hippocampal amyloid β (Aβ) in wild type (WT) mice and long-term sleep loss increases amyloid plaque in AD transgenic mouse models. Both effects can be influenced by the wake-promoting neuropeptide, hypocretin (HCRT), but whether HCRT influences amyloid accumulation independent of sleep and wake timing modulation remains unclear. Here, we induced chronic fragmentation of sleep (CFS) in WT and HCRT-deficient mice to elicit similar arousal indices, sleep bout lengths and sleep bout numbers in both genotypes. We then examined the roles of HCRT in CFS-induced hippocampal Aβ accumulation and injury. CFS in WT mice resulted in increased Aβ_42_ in the hippocampus along with loss of cholinergic projections and loss of locus coeruleus neurons. Mice with HCRT deficiency conferred resistance to CFS Aβ_42_ accumulation and loss of cholinergic projections in the hippocampus yet evidenced similar CFS-induced loss of locus coeruleus neurons. Collectively, the findings demonstrate specific roles for orexin in sleep disruption hippocampal injury.

**Significance statement:**

Chronic fragmentation of sleep (CFS) occurs in common conditions, including sleep apnea syndromes and chronic pain disorders, yet CFS can induce neural injury. Our results demonstrate that under conditions of sleep fragmentation, hypocretin/orexin is essential for the accumulation of amyloid-β and loss of cholinergic projections in the hippocampus observed in response to CFS yet does not influence locus coeruleus neuron response to CFS.

## Introduction

Chronic fragmentation of sleep (CFS) occurs in many prevalent conditions, including obstructive sleep apnea and chronic pain disorders, and yet, CFS may negatively impact brain health. The presence of CFS in cognitively normal older individuals increased the likelihood of developing Alzheimer’s disease (AD); notably, the more frequently sleep was disturbed, the greater the decline in cognitive performance (Lim et al., 2013). In a separate study, CFS in older adults predicted reduced metabolism in the hippocampus (Andre et al., 2019), supporting hippocampal vulnerability to CFS. In wild-type (WT) mice, CFS resulted in several pathological changes consistent with AD, e.g., increased phosphorylation of tau in the hippocampus and upregulation of microglial and astrocyte reactivity markers in the hippocampus and cortex (Xie et al., 2020; Ba et al., 2021). CFS in adult rats reduced hippocampal neurogenesis and impaired spatial (hippocampal-dependent) memory performance (Sportiche et al., 2010), and CFS in the APP/PS1 mouse transgenic model of AD pathology increased amyloid plaque burden in the hippocampus and cortex (Minakawa et al., 2017) and increased both amyloid-β_42_ (Aβ_42_) and markers of neuroinflammation in the hippocampus but not in the cortex (Duncan et al., 2022). These studies demonstrate in multiple species that CFS promotes amyloid-β dyshomeostasis and that the hippocampus is vulnerable to CFS.

How CFS disturbs neural processes in the hippocampus is not known. There is some evidence in transgenic AD mouse models that the hypothalamic neuropeptide hypocretin (HCRT), also known as orexin, might contribute to both neuroinflammation and β-amyloid accumulation. Specifically, intracerebroventricular administration of HCRT acutely increases Aβ peptide levels in the interstitial space of the hippocampus and cortex, while acute administration of a HCRT antagonist to AD transgenic mice lowers Aβ peptide levels, and chronic HCRT antagonism reduces amyloid plaque burden (Kang et al., 2009). Because HCRT administration increases wake and its antagonism promotes sleep, it remains unclear whether the HCRT effects on Aβ are specific to HCRT or occur secondarily through disruption of sleep. When transgenic AD mice with *HCRT* deficiency were chronically deprived of sleep 18 h/day, amyloid plaque burden increased (Roh et al., 2014), supporting HCRT-independent effects of severe sleep disruption on amyloid plaque in an AD transgenic model, but whether HCRT is necessary for the sleep disruption-induced increases in Aβ and neural injury in WT mice is not known. Furthermore, while HCRT may not be necessary for sleep loss-induced amyloid plaque in AD transgenic mice, HCRT may play a role in other early pathologic manifestations in AD, for example loss of cholinergic fibers in the hippocampus (Stokin et al., 2005), which may contribute to impaired cognition in AD (Hampel et al., 2018). In support of a direct HCRT effect on Aβ homeostasis, there is evidence that HCRT has direct effects on both microglial phagocytosis of Aβ and on amyloid precursor protein (APP) processing in the hippocampus that support roles for HCRT in amyloid dyshomeostasis (Ma et al., 2016; An et al., 2017; Li et al., 2020).

Here we developed a model of comparable sleep fragmentation in HCRT-deficient and WT mice and then explored the role of endogenous HCRT in hippocampal Aβ and cholinergic axonal injury in response to CFS. We found that CFS in WT mice resulted in a loss of hippocampal cholinergic projections and increased hippocampal punctate Aβ_42_ immunoreactivity. We also confirmed that CFS results in a loss of locus coeruleus neurons (LCn) in WT mice. HCRT−/− mice conferred resistance to CFS loss of hippocampal cholinergic fibers and increased Aβ, yet these mice were equally susceptible to CFS-induced loss of LCn. Collectively these new findings support the concept that HCRT contributes to CFS hippocampal injury, CFS LCn injury appears to occur independent of HCRT signaling.

## Materials and methods

### Chronic fragmentation of sleep protocols

Studies were performed at the University of Pennsylvania in accordance with the National Institutes of Health Office of Laboratory Animal Welfare Policy and the Institutional Animal Care and Use Committee. Male and female C57BL/6J and *hypocretin* knockout mice on a B6 background (B6.129S6-*Hcrt*^*tm*1Y*wa*^/J, *HCRT*−/− mice) from the Jackson Laboratory mice were studied. Mice were 3–4 months old at the start of CFS or Rest control conditions. CFS was performed using a method developed by Sinton et al. (2009) to jostle the cages of sleep deprived mice intermittently. Specifically, CFS cages, were placed atop an orbital rotor (MaxQ 2000) with the speed set at 120 rotations per minute (RPM), which was on for 10 s every minute and then off for the remaining 50 s of each minute for 24 h/day, controlled by a timer (H3CR-F8-300, Omron). Rested controls were housed in the same room but were not exposed to cage movement. CFS was administered for 10 weeks. All mice were returned to their colony for 1 month prior to histological evaluation (7–8 months of age). Throughout the study, including CFS exposures, mice were maintained on the same 12:12 h light:dark schedule and fed *ad libitum* standard rodent chow and water. Long water nozzles with ball valves were used to prevent leaking.

### Electrode implantation and sleep recording and analysis

To assess the effectiveness of the CFS protocol on sleep in the two genotypes, WT and HCRT−/− mice were implanted with brain surface electroencephalographic (EEG) and dorsal nuchal electromyographic (EMG) electrodes to identify behavioral states. Electrodes were fashioned from perfluoroalkoxy-coated silver wire (787000, A-M Systems, diameter 127 μm) by melting the proximal electrode tip to form a uncoated sphere (diameter 0.5 mm) for electrical signal contact. General anesthesia was induced with 3–4% isoflurane via mask, and then maintained with 1–2.5% isoflurane. Using sterile procedures, 0.5 mm diameter holes were drilled in the skull surface for placement of insulated silver EEG electrodes just below the skull surface, yet not piercing the dura (ML 1.5 mm; AP-1.8 mm, relative to Bregma) bilaterally, and reference electrodes were implanted rostrally just below the skull surface (ML 2.0 mm, AP + 1.5 mm, relative to Bregma) bilaterally. Nuchal electromyographic (EMG) electrodes were sutured into the dorsal nuchal musculature. All electrode wires were attached to a connector pedestal (MS363, Plastics One) which was secured with dental acrylic (8101, 8501, Pearson Dental). Mice recovered with littermates for 5 days and were then placed in individual cages, prior to connecting the recording cable and commutator (363 SL/6, SL6C Plastics One), for 1 week before recordings were obtained. To ascertain how the CFS protocol fragmented sleep in the two genotypes, sleep/wake recordings were obtained in CFS mice during week 3 of CFS (to allow some acclimation to CFS), and control mice were recorded at the same time (*n* = 5/genotype, sleep condition).

EEG and EMG signals were acquired at 256 Hz sampling frequency and amplified and filtered (15A94 Grass Technologies). EEG signals were filtered with 0.5–30 Hz and EMG with 1–100 Hz cut-off frequencies. Data were recorded on AcqKnowledge v.3 software and analyzed on SleepSign 3.2., Kissei Comtec. Data were binned into 4-s epochs for 24 h (Zeitgeber 0–24) and each epoch was scored as Wake, non-rapid eye movement sleep (NREMS) or rapid eye movement sleep (REMS). Wake epochs were defined as epochs with low amplitude, desynchronized EEG activity with higher EMG activity; NREMS epochs were defined when > 30% of EEG waveforms within the epoch showed slow wave (0–4 Hz) activity, and REMS epochs were defined when > 30% of the EEG activity was of theta frequency (6–10 Hz) (Li et al., 2014). The sleep analysis software outlined detected slow wave and theta activity in each epoch, and all epochs were scored manually by scorers blinded to sleep condition and genotype. The primary variable was the arousal frequency, which was defined the frequency per hour of sleep of the occurrence of one or more consecutive wake epochs following five or more sleep epochs (NREMS and/or REMS epochs). Wake bouts were defined as > 4 epochs of wakefulness after > 4 epochs of sleep. NREMS bouts were defined as > 4 epoch of NREMS after > 4 epochs of wake or REMS, and REMS bouts were defined as > 3 REMS epochs after > 4 epochs of either wake or NREMS. Additional sleep analyses were limited to percentage time in each stage, the average duration of bouts in each behavioral state and the number of bouts in each behavioral state/24 h.

### Histology, microscopy, and stereology

At 7–8 mos of age, 1 month after CFS or rested (Rest) exposures, mice were anesthetized with intraperitoneal sodium pentobarbital and transcardially perfused with 4% paraformaldehyde. Brains were cryopreserved and then coronally sectioned at 60 μm. Sequential sections were placed in 1:6 series to allow for stereology (Panossian et al., 2011). For immunohistology, selected sections were washed and blocked in 0.1% Triton-PBS-1%BSA with 1:50 mouse IgG added for mouse primary antibodies. Sections were then incubated with primary antibodies diluted in blocking buffer for 1–3 days at room temperature and/or 4°C. Primary antibodies used were: *Aβ42* (C-term), AB5078P Millipore; *Aβ42* (12F4), 805501 Biolegend; *Glial fibrillary acidic protein, (GFAP)*, 13-0300 Thermo Fisher Scientific; *Ionized calcium-binding adapter molecule-1 (Iba-1)*, Ab107159 Abcam; *Tyrosine hydroxylase* (*TH*) LS-C124752 LSB and *Vesicular acetylcholine transferase (VAchT)* 139103, SynSystems. Sections from APP^–/–^ (B6.129S7 *APP^tmidbo^*/J mice were used (Jackson Laboratory) to confirm Aβ antibody specificity and to optimize confocal settings for detection Aβ_42_ with minimal non-specific labeling. Sections processed without primary antibodies were used to normalize for non-specific labeling and autofluorescence. For light microscopy immunohistology (locus coeruleus stereology), secondary antibodies were labeled with Vector blue alkaline phosphatase and counter-stained in Giemsa to allow chromatin visualization for stereological counts. For confocal imaging, secondary antibodies were conjugated with Alexa Fluor probes: 488, 555, 594, or 647 (Invitrogen). Imaging was performed with Leica DM5500B (light microscopy) and DM4B (stereology) and Leica SP5/AOBS (confocal). Confocal laser intensities, ηm range, detector gain, exposure time, amplifier offset, and depth of the focal plane were standardized across compared sections (Panossian et al., 2011). For Aβ_42_ image acquisition, Aβ_42_ immunolabeled hippocampal sections from the APP−/− mouse were used to set settings for zero signal. VAchT axonal projection density within the CA1 hippocampus was measured as the% area VAchT fibers across CA1 in each mouse (Zhu et al., 2016). Confocal images for Aβ_42_, Iba-1, GFAP, VAchT were obtained in the CA1 region of three sections per mouse between Bregma −1.7 to −2.7 mm. Images (40 × 2) were obtained across 4 mm of tissue at the same depth for each animal and below a 2 mm guard zone. Fluorescent image analysis for each primary antibody target was performed using ImageJ software by converting images of single color to 8-bit grayscale inverted images. A detection threshold was standardized across all images for a given primary antibody target, and the percent area of labeling within CA1 was determined as the primary variable and averaged per animal.

Total LCn count estimations of TH antibody labeled neurons with Giemsa stained nuclei were performed using optical fractionator stereology (West and Gundersen, 1990), using 1:2 series of sections (LC sections in wells 1, 3, and 5), covering Bregma −5.02 to −5.80 mm) for both genotypes and sleep conditions (*n* = 5–6/group), as described in detail (Zhang et al., 2014). Stereology was performed using the Leica DM4B microscope with a StereoInvestigator workstation (MicroBrightField, v.11.09) (West and Gundersen, 1990). A 100x oil objective was used to count cells with focused chromatin within the probe boundaries with TH-labeled cytoplasmic diameters > 15 um. A sampling scheme with 0.25 area sampling fraction and 0.80 thickness sampling fraction was used. This strategy provided > 200 counts/mouse across 8–9 sections and Gundersen coefficients of error < 0.10. Scorers were blinded to age and sleep conditions.

### Statistical analysis

Statistical analyses were performed using GraphPad statistical software (Prism, version 6.0). Dataset groups were tested for normality using the Kolmorgorov-Smirnov normality test. For data that passed normality, two-way ANOVA was used to assess sleep condition and genotype effects. Where overall differences or main (sleep or genotype) effects were observed, Sidak’s *post hoc* analyses were used for selected individual multiple comparisons. The predetermined *post hoc* comparisons were sleep condition effects within each genotype and genotype effects within each sleep condition. For normal single comparisons of normal data, an unpaired *t*-test was used. For data that did not pass the normality test, a Kruskal-Wallis one-way ANOVA with Dunn’s multiple comparisons was performed across the four groups. The cutoff for significant statistical power for all analyses was a Sidak’s or Dunn’s corrected *p* < 0.05.

## Results

### Hypocretin−/− mice show an abnormal circadian distribution of wake and shortened wake periods in the active period

**FIGURE 1:**
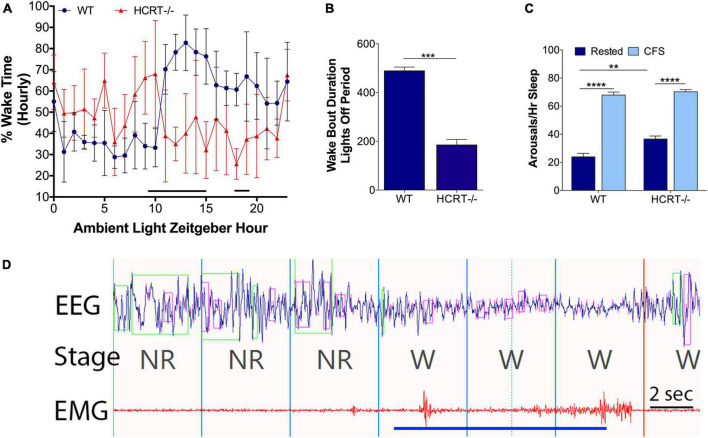
Behavioral state effects of HCRT deficiency and CFS effects on arousal frequency in HRCT–/– and WT mice. Sets of mice of both studied genotypes (wild type, WT and hypocretin-deficient, HCRT–/–) and both sleep conditions, rested and chronic fragmentation of sleep (CFS) underwent 24 h electroencephalography (EEG) and electromyography (EMG) recordings to confirm baseline sleep/wake abnormalities in HCRT–/– mice and to determine the effectiveness of rotor platform movement on sleep consolidation. **(A)** Hourly percent (%) wake time for Rest WT (blue dots) and Rest HCRT–/– (red triangles) mice (mean ± SE, *n* = 5/group). Repeated measures ANOVA identified differences at Zeitgeber hours 10–16 and 18 and 19 (black line). **(B)** Group data for Rest WT and HCRT–/– mice for the average duration of wake bouts in the dark (lights-off) period expressed in minutes (mean ± SE) analyzed with an unpaired *t*-test, ***p < 0.001. **(C)** Number of arousals/h of sleep (averaged across 24 h) group data (mean ± SE, *n* = 5/group) for WT and HCRT–/– mice exposed to Rested (dark blue) or chronic fragmentation of sleep (CFS, light blue), *n* = 5/group. Data were analyzed as two-way ANOVA with selected *post hoc* comparisons (Sidak’s test), **p < 0.01; ****p < 0.0001. **(D)** A representative raw tracing of the effects of rotor table movement on the EEG and EMG signals in a mouse. Rotor-elicited arousals looked similar in both WT and HCRT–/– mice. The blue line depicts the time of the shaker table movement. 4-s epochs, demarcated with vertical lines in the fragment, were scored as non-rapid-eye-movement sleep (NR) and wakefulness (W). Time bar, 2 s. Over the EEG tracing, green boxes denote computerized program detection of delta waves (0–4 Hz), and pink boxes denote computer detection of theta waves (6–10 Hz).

A finding in humans and mouse model narcolepsy is an alteration in the 24-h distribution of sleep wake times (Chemelli et al., 1999; Lin et al., 1999; Hara et al., 2001; Wurtman, 2006; Nishino, 2007). 24-h hourly% time spent in wake was analyzed for WT and HCRT−/− mice with repeated measures ANOVA and Sidak’s multiple comparison analysis corrected for 24 time points. As expected, there was an overall interaction, *F*_(23, 184)_ = 7.2, *p* < 0.00001. There were both time of day interactions (*F* = 3.0, *p* < 0.0001) and genotype interactions (*F* = 12.1, *p* < 0.01). Significant genotype differences were observed for Zeitgeber hours 9–15 and 18 and 19 (t’s = 3.1–4.6, p’s < 0.05), as summarized in Figure 1A. A shortening of wake bouts in the active period is also observed in narcolepsy (Chemelli et al., 1999; Nishino, 2007). Wake bouts in the lights-off period were analyzed with *t*-tests and were shortened in HCRT−/− mice (*t* = 11.3, *p* < 0.001), as summarized in Figure 1B.

### Intermittent rotor platform increases the arousals frequency in both wild type and hypocretin−/− mice

An overall interaction for the number of arousals/hour of sleep (arousal index) for the 24 h cycle was observed, *F*_(1, 16)_ = 6.1, *p* < 0.05, as summarized in Figure 1C, with differences observed for genotype (*F* = 13.0) and sleep conditions (*F* = 342.0). The arousal index was increased in CFS WT mice, relative to Rest WT mice (Sidak’s *t* = 14.8, *p* < 0.0001) and in CFS HCRT−/− mice, relative to Rest HCRT−/− mice (*t* = 11.3, *p* < 0.0001). The arousal index in rested HCRT−/− was higher than in rested WT−/− mice (*t* = 4.3, *p* < 0.01), and in mice exposed to CFS, the arousal index was similar across the two genotypes (*t* = 0.8, N.S). A representative electrographic platform rotor-elicited brief arousal is shown in Figure 1D. In summary, with this CFS paradigm, the frequency of sleep disruption was equivalent across genotypes.

### Genotype and chronic fragmentation of sleep effects on behavioral state

**FIGURE 2:**
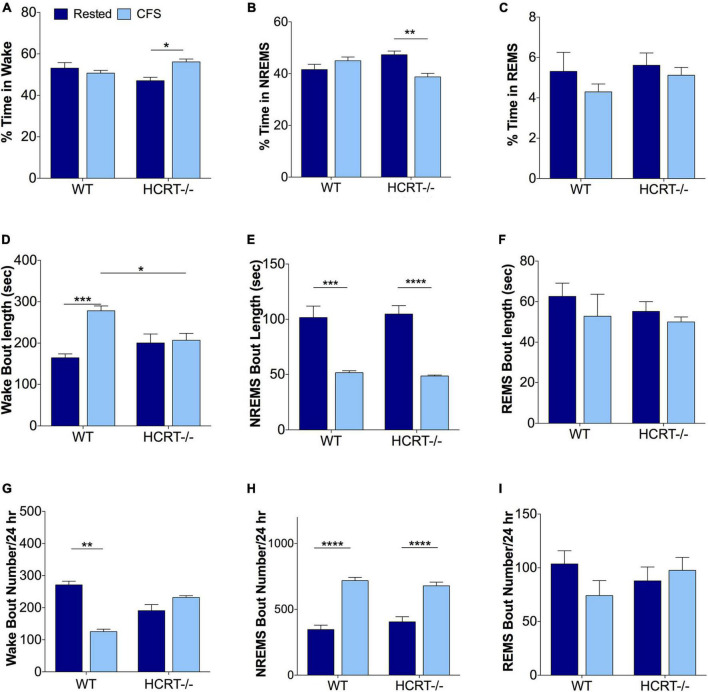
Sleep disturbances from chronic sleep fragmentation are NREMS specific for both genotypes. **(A–C)** Percentages of time/24 h spent in Wake **(A)**, NREMS **(B)**, and REMS **(C)**. Data are presented as mean ± SE (*n* = 5/group), for Rested, dark blue and CFS, light blue across the two genotypes. **(D–F)** Behavioral state bout lengths expressed as mean ± SE, and **(G–I)** bout numbers/24 h for the three behavioral states. Data in **(A–E,H,I)** were analyzed as two-way ANOVA, corrected for selected *post hoc* comparisons (Sidak’s test). Non-normal data in **(F,G)** were analyzed with Kruskall-Wallis and Dunn’s *post hoc* comparisons. *p < 0.05; **p < 0.01; ***p < 0.001; ****p < 0.0001.

We have shown previously in WT mice that this mode of chronic sleep disruption results in both an increased arousal index and shortened periods of NREMS (Li et al., 2014). Here, we assessed the effects of platform movement on 24 h values for the% time spent in each behavioral state, the average length of bouts in each state and the number of bouts in each state in WT and HCRT−/− mice. An overall interaction was observed for% wake time, *F*_(1, 16)_ = 9.8, *p* < 0.01. CFS effects on% wake time per 24 h are summarized in Figure 2A. The only significant *post hoc* comparison for wake% was that CFS increased wake% time in HCRT−/− mice, relative to rest HCRT−/− mice wake% time, *t* = 3.5, *p* < 0.05. Similarly, an overall interaction was observed across the same groups for NREMS (*F* = 14.2, *p* < 0.01, Figure 2B), where a CFS effect on NREMS% time/24 h was observed only for HCRT−/− mice, where% NREMS was reduced in CFS mice, relative to Rest mice (*t* = 5.4, *p* < 0.01). No genotype or sleep condition interactions were observed for% REMS/24 h (Overall F = 0.2, N.S., Figure 2C).

Differences in wake bout lengths were observed *F*_(1, 16)_ = 11.8, *p* < 0.01, as summarized in Figure 2D. The average length of wake bouts/24 h did not differ with genotype for rested conditions (*t* = 2.3, N.S.), but lengthened in WT mice in response to CFS, relative to WT Rested, *t* = 7.3, *p* < 0.001, and wake bout lengths were longer in WT CFS relative to HCRT−/− CFS, *t* = 4.6, *p* < 0.05. In contrast, CFS did not affect wake bout length in HCRT−/− mice, *t* = 0.4, N.S. An overall interaction was not observed for NREMS bout lengths, *F*_(1, 16)_ = 0.2, N.S., but a sleep condition effect was observed, *F* = 67.3, *p* < 0.0001. *Post hoc* analyses showed reductions in NREMS bout lengths (*t* = 7.7, *p* < 0.001) in both the WT (*t* = 7.7, *p* < 0.001) and HCRT−/− mice (*t* = 8.7, *p* < 0.0001) in CFS, relative to Rest mice. Notably, there were no differences in NREMS bout lengths in CFS WT and CFS HCRT−/− mice, *t* = 0.4, N.S., Figure 2E). REMS bout length data did not pass normality testing and were analyzed with Kruskal-Wallis one way ANOVA. There were no differences across the four groups, (K-W = 3.4, N.S., Figure 2F). Thus, the rotor platform paradigm effectively shortened NREMS bouts in mice of both genotypes to similar average lengths without seeming to impact REMS bout duration.

Wake bout numbers did not pass normality data and were analyzed with Kruskal-Wallis one-way ANOVA with Dunn’s *post hoc* comparisons. An overall difference was observed across the four groups (K-W = 15.4, *p* < 0.01, Figure 2G). Individual comparisons revealed a difference only for Rest WT relative to CFS WT, *p* < 0.001. While no overall interaction was observed for NREMS bout numbers, *F*_(1, 16)_ = 2.4, N.S., a strong a sleep condition effect was observed, *F* = 105, *p* < 0.0001. *Post hoc* comparisons, as summarized in Figure 2H, revealed that NREMS bout numbers in WT mice increased in response to CFS (*t* = 11.8, *p* < 0.0001) and in HCRT−/− mice in response to CFS (*t* = 8.7, *p* < 0.0001). There were no genotype effects across Rest conditions (*t* = 1.8, N.S.) or in CFS conditions (*t* = 1.2, N.S.). REMS bout numbers were changed by neither sleep condition nor genotype, *F*_(1, 16)_ = 2.3, N.S, Figure 2I. In summary, CFS was effective in both strains of mice in disrupting NREMS so that both genotypes had similar numbers of NREMS bouts and bout lengths in response to CFS. In contrast, REMS appeared unperturbed by CFS.

### Chronic fragmentation of sleep increases Aβ_42_ in the hippocampus in wild type but not hypocretin−/− mice

**FIGURE 3:**
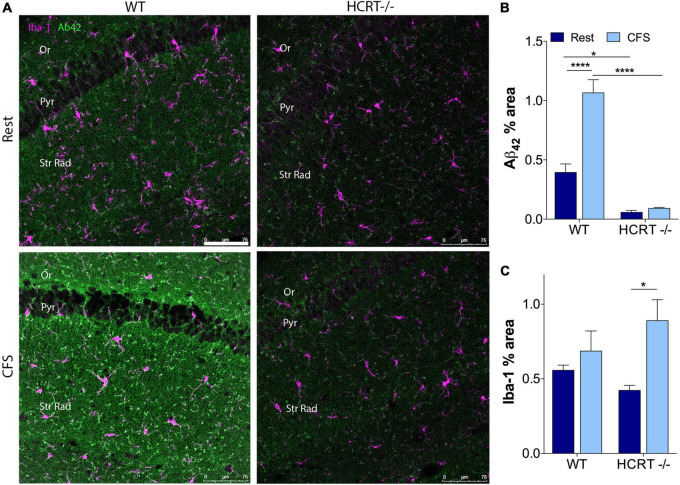
HCRT–/– mice confer resistance to CFS Aβ_42_ accumulation. **(A)** Representative confocal images within the CA1 hippocampus across the genotype and sleep conditions immunolabeled for Aβ_42_ (green) and microglial marker Iba-1 (purple). Calibration bar, 75 μm. Subregions of CA1 are labeled: Oriens, Or; pyramidal neuron somata, Pyr; and Strata Radiatum, Str Rad. **(B)** Group data for percentage area CA1 Aβ_42_, expressed as mean ± SE (*n* = 5–7/group) for Rested (dark blue) and CFS (light blue) for WT and HCRT–/– mice. **(C)** Group data for percentage area CA1 Iba-1, expressed as mean ± SE (*n* = 5–7/group) for Rested (dark blue) and CFS (light blue) for WT and HCRT–/– mice, analyzed as two-way ANOVA for sleep condition and genotype with *post hoc* correction, Sidak’s. *p < 0.05; ****p < 0.0001.

To determine whether orexin influences sleep-disruption accumulation of Aβ_42_ in the hippocampus, we examined Aβ_42_% area within CA1 across the sleep conditions and genotypes. An overall interaction was observed for Aβ_42_ in the hippocampus, *F*_(1, 12)_ = 12.9, *p* < 0.01. Across Rest mice, Aβ_42_ was higher in WT than in HCRT−/− mice (*t* = 4.6, *p* < 0.05). As observed in response to chronic short sleep, CFS in WT mice resulted in an increase in Aβ_42_ in a punctate pattern (*t* = 7.6, *p* < 0.001, Figures 3A,B). In contrast, in HCRT−/− mice, Aβ_42_ did not increase in response to CFS, *t* = 0.4, N.S. Consequently, hippocampal Aβ_42_ was elevated in WT CFS mice, relative to HCRT−/− CFS mice, *t* = 11.8, *p* < 0.0001. In summary hippocampal Aβ_42_ was higher in Rest WT than in rest HCRT−/− mice and increased only in WT mice in response to CFS.

### Chronic fragmentation of sleep and hypocretin influence hippocampal glia

CFS has been shown to increase microglial Iba-1 and astrocyte GFAP responses in the hippocampus in WT mice (Ba et al., 2021). Here, we examined whether HCRT influences the glial responses to CFS. There was no overall interaction for CA1 microglial Iba-1, *F*_(1, 16)_ = 2.6, N.S., but a main sleep effect was observed, *F* = 8.8, *p* < 0.01, as summarized in Figure 3C. Specifically, *post hoc* analysis revealed an increase in Iba-1% area in HCRT−/− mice exposed to CFS, relative to Rest HCRT−/− mice (*t* = 4.7, *p* < 0.05), but this% area in CFS HCRT−/− mice did not differ from CFS WT mice (*t* = 2.0, N.S.). There was no overall interaction for genotype and sleep condition for GFAP, *F*_(1, 16)_ = 3.7, N.S. and no genotype or sleep main effects, *F* = 0.05, N.S. and *F* = 4.1, N.S., respectively. Data are not shown. Overall, there were no genotype effects with microglia or astrocytes to explain the genotype differences in Aβ.

### Loss of hypocretin protects hippocampal cholinergic projections loss from chronic fragmentation of sleep

**FIGURE 4:**
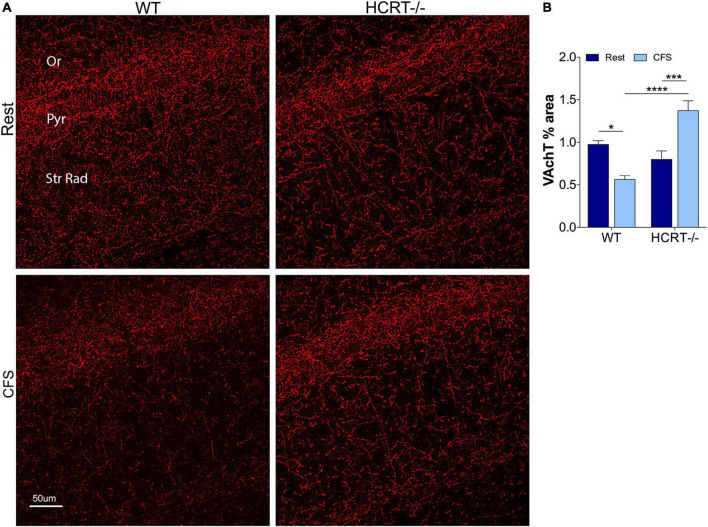
HCRT-deficient mice confer resistance to CFS-induced loss of cholinergic projections in the CA1 region of the hippocampus. **(A)** Representative confocal images of vesicular acetylcholine transporter (VAchT, red) immunoreactivity in CA1 hippocampus across the genotype and sleep conditions. Subregions of CA1 are labeled: Oriens, Or; pyramidal neuron somata, Pyr; and Strata Radiatum, Str Rad. Calibration bar, 50 μm. **(B)** Group data for percentage area CA1 VAchT, expressed as mean ± SE (*n* = 5–7/group) for Rest (dark blue) and CFS (light blue) for WT and HCRT–/– mice. Data were analyzed with two-way ANOVA and Sidak’s *post hoc* tests, *p < 0.05; ***p < 0.001; ****p < 0.0001.

In light of the Aβ differences across HCRT genotypes, we next examined whether HCRT presence influences the cholinergic projections into CA1 hippocampus by examining% coverage of VAchT immunoreactive axons across sleep and genotype groups. An overall interaction was observed, *F*_(1, 16)_ = 6.4, *p* < 0.05, and main sleep condition effects were found (*F* = 23.22, *p* < 0.0001). In WT mice, CFS reduced the VAchT area% (*t* = 6.0, *p* < 0.0001, Figures 4A,B), and in contrast, in HCRT−/− mice CFS did not reduce VAchT fiber% area (*t* = 1.4, N.S.), and in CFS mice, VAchT% area was higher in HCRT−/− than in WT mice (*t* = 3.0, *p* < 0.05). In summary, cholinergic projections into the hippocampus were reduced in WT CFS, relative to WT Rest mice, while cholinergic projections were protected from CFS in HCRT−/− mice.

### Hypocretin genotype did not influence chronic fragmentation of sleep effects on locus coeruleus neurons

**FIGURE 5:**
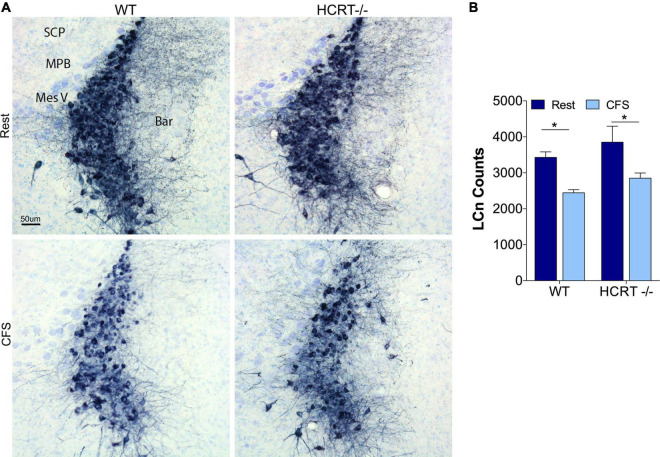
HCRT genotype-independent effects of CFS on locus coeruleus neuron counts. **(A)** Representative mid-locus coeruleus (LC) coronal sections immunolabeled for tyrosine hydroxylase detected with substrate blue (navy) and counterstained with Giemsa staining across the four groups. Surrounding landmarks: Superior Cerebellar Peduncle, SCP; Medial Parabrachialis nucleus (MPB; Mesencephalic Trigeminal, Mes V; and Barrington’s nucleus, Bar. Calibration bar, 50 μm. **(B)** Group data for stereological LC cell counts, expressed as mean ± SE (*n* = 5/group) for Rest (dark blue) and CFS (light blue) for WT and HCRT–/– mice, analyzed with two-way ANOVA and Sidak’s *post hoc* tests, *p < 0.05.

We have shown previously that CFS results in injury to locus coeruleus neurons (LCn) that manifests, in part, with reduced numbers of LCn (Zhu et al., 2015). Here we examined whether CFS-induced LCn loss is influenced by the presence of HCRT. A main effect of sleep condition was observed, *F*_(1, 16)_ = 13.4, *p* < 0.01, Figures 5A,B. CFS reduced the LCn counts in both WT (*t* = 4.2, *p* < 0.05) and HCRT−/− mice (*t* = 4.1, *p* < 0.05), and there were no genotype effects observed for wither Rest (*t* = 1.7, N.S.) or CFS conditions (*t* = 1.7, N.S.). In summary, CFS effectively reduced LCn counts in both genotypes, and no genotype effect was observed for LCn counts under either Rest or CFS conditions.

## Discussion

Chronic disruption of sleep, including CFS, increases Aβ in the brain, particularly in the hippocampus (Roh et al., 2012; Qiu et al., 2016; Minakawa et al., 2017; Zhao et al., 2017; Owen et al., 2021; Duncan et al., 2022). Yet the molecular mechanisms by which sleep disruption influences Aβ production and/or clearance are largely unknown. In the present work, we confirm that CFS increases Aβ at least in the CA1 region of the hippocampus. The increase in hippocampal Aβ occurred with specific fragmentation of NREMS, as REMS bout durations appeared undisturbed. We extended the phenotype of CFS hippocampal injury to include loss of cholinergic projections, and we now provide evidence that HCRT influences CFS-induced Aβ_42_ accumulation and loss of septohippocampal cholinergic fibers. Additionally, we demonstrate that HCRT deficiency does not influence LCn survival in response to CFS.

Earlier observations that acute administration of HCRT increases brain Aβ and that HCRT antagonism lessened amyloid plaque accumulation in a mouse model of AD (Kang et al., 2009) prompted assessment of the prevalence of AD in humans with narcolepsy. In an early study, brains of elderly individuals (mean age, 86 years) with narcolepsy with cataplexy (Type I) were examined for AD pathology (Scammell et al., 2012). The study found a similar prevalence of AD pathology in the brains of individuals with Type I narcolepsy, as expected in the general population (Scammell et al., 2012). However, a more recent study directly compared brain amyloid burden (assessed with ^18^florbetapir positron emission tomography imaging) in a somewhat younger population of Type I narcoleptics (>65 years, with most individuals < 80), with confirmed low cerebrospinal fluid HCRT levels and age-matched non-narcoleptic individuals (Gabelle et al., 2019). They found less amyloid plaque burden in narcoleptic participants than in two distinct age-matched control groups (Gabelle et al., 2019). Collectively, these studies support the concept that loss of HCRT neurons may slow or delay Aβ plaque accumulation yet may not prevent the ultimate development of amyloid plaque accumulation in individuals with sufficiently long lifespans. Additionally, from the human narcolepsy amyloid studies, it remains unclear whether the delay in amyloid accumulation is the consequence of loss of HCRT or of other neurotransmitters that are also released from HCRT neurons (Chou et al., 2001; Torrealba et al., 2003). Here, we found that Rest HCRT−/− middle-aged mice showed significantly less Aβ than Rest WT mice, without having reduced total amounts of wakefulness or increased NREMS for the 24 h cycle. It is possible that diurnal differences in sleep/wake patterns in Rest HCRT−/− and Rest WT mice influence Aβ, but this would suggest that seemingly small differences in normal (WT) sleep/wake timing can influence hippocampal Aβ. CFS effectively fragmented both WT and HCRT−/− mice across the 24 h cycle, and yet only HCRT−/− mice conferred resistance to CFS-hippocampal Aβ accumulation. Thus, CFS in WT mice increases Aβ_42_, while in HCRT−/− mice a similar frequency of CFS does not. Collectively, our findings support a role for HCRT in determining hippocampal Aβ homeostasis in both Rest and CFS conditions. Thus, HCRT may influence Aβ_42_ production and/or clearance.

There are several behavioral state-independent mechanisms whereby HCRT could influence Aβ production and/or clearance. Microglial cells play major roles in the uptake and clearance of both soluble and fibrillar Aβ (Lee and Landreth, 2010). HCRT applied to microglial cells in culture reduces the glial cells’ motility (which may inhibit Aβ uptake and clearance) and suppresses degradation of Aβ (An et al., 2017). Additionally, HCRT exogenously applied to a neural cell line (SH-SH5Y cells) in culture can impart mitochondrial dysfunction and injury and increases oxidative stress (Li et al., 2020; Zhu et al., 2021), which may then promote Aβ production (Plascencia-Villa and Perry, 2021). HCRT-1 (orexin-A) peptide upregulates calcium/calmodulin-dependent kinase II (CaMKII), which can then phosphorylate tau and modulate Aβ synaptotoxicity (Oka et al., 2017; Opazo et al., 2018; Fan et al., 2021). Whether HCRT impairs CFS microglial function and whether CFS increases HCRT to levels required to induce mitochondrial injury and/or CAMKII activation should now be examined.

Degeneration of cholinergic neurons is a prominent finding in AD, and loss of cholinergic neurons predicts cognitive impairment (Geula et al., 1998; Mesulam et al., 2004). In the present study, the effects of CFS on cholinergic projections into the hippocampus diverged for HCRT−/− and WT mice. Specifically, CFS reduced the density of cholinergic projections in WT mice, yet not in HCRT−/− mice. HCRT neurons project widely throughout the brain, to include dense innervation of the basal forebrain (Peyron et al., 1998). Arousal from sleep increases the activity of HCRT neurons (Takahashi et al., 2008), and thus likely output of HCRT. Ultrastructural studies have demonstrated direct HCRT bouton-cholinergic neuron synapses in the medial septum of the basal forebrain (Wu et al., 2004). Local application of HCRT peptides directly activates basal forebrain cholinergic neurons and increases acetylcholine release in the hippocampus in young adult rats (Eggermann et al., 2001; Stanley and Fadel, 2012). Aβ production is linked to neuronal activity (Bero et al., 2011). Therefore, Aβ production is expected to directly increase upon activation of both HCRT and cholinergic neurons, and hippocampal targets of cholinergic septohippocampal projections. We propose that CFS-HCRT signaling imposes significant metabolic challenges on medial septal cholinergic neurons by increasing neuronal activation through disruption of sleep.

In turn, loss of cholinergic innervation can alter Aβ homeostasis (Gil-Bea et al., 2012; Laursen et al., 2013; Ramos-Rodriguez et al., 2013; Hartig et al., 2014; Turnbull et al., 2018). Specifically, degeneration of basal forebrain cholinergic neurons in mouse models of AD increases Aβ production and/or accumulation in the forebrain, including the hippocampus, where the Aβ increase can include both soluble Aβ_42_ and amyloid plaque (Gil-Bea et al., 2012; Laursen et al., 2013; Ramos-Rodriguez et al., 2013; Hartig et al., 2014; Turnbull et al., 2018). Basal forebrain cholinergic neurons are unique for the presence of the 75-kD neurotrophin receptor (p75NTR), and this receptor can bind and internalize Aβ and promote Aβ lysosomal clearance (Ovsepian et al., 2014). Thus, in CFS WT mice, the reduction in septohippocampal cholinergic fibers may contribute to the CFS increase in hippocampal Aβ_42_. Notably a feed-forward cycle of CFS neural injury may have developed, as loss of septohippocampal cholinergic neurons can increase Aβ, and increased Aβ would then injure cholinergic neurons.

Locus coeruleus are susceptible to substantial loss in AD (Oh et al., 2019), and most, but not all, studies have demonstrated loss of LCn in response to sleep disruption (Shaffery et al., 2012; Li et al., 2014; Zhang et al., 2014; Zhu et al., 2015; Deurveilher et al., 2021). HCRT fibers project densely to the LCn (Peyron et al., 1998), and HCRT-mediated arousals require LCn activation (Carter et al., 2012). Despite robust CFS genotype effects for Aβ and cholinergic projection effects in the hippocampus, LCn were equally vulnerable to loss in both WT and HCRT−/− mice, suggesting that the CFS injury to LCn is not modified by HCRT, despite the dense projections (Peyron et al., 1998). Additionally, with comparable loss of LCn in both genotypes, the present findings also provide evidence that loss of LCn is not a major determinant in hippocampal injury.

In conclusion, chronic sleep disruption through fragmentation of NREMS increases Aβ and reduces septocholinergic projections in the hippocampus, and HCRT deficiency is effective in preventing both CFS-induced effects. CFS also results in loss of LCn, but this effect is unaffected by of the presence of HCRT. The work provides evidence of important HCRT-dependent and -independent processes in CFS neural injury.

## Data availability statement

The original contributions presented in this study are included in the article/supplementary material, further inquiries can be directed to the corresponding author/s.

## Ethics statement

The animal study was reviewed and approved by the University of Pennsylvania IACUC.

## Author contributions

SV presented hypotheses and designed studies. HN, PF, and YZ conducted the studies and analyzed the data. HN and SV wrote the manuscript. All authors contributed to the article and approved the submitted version.

## References

[B1] An H. Cho M. H. Kim D. H. Chung S. Yoon S. Y. (2017). Orexin impairs the phagocytosis and degradation of amyloid-beta fibrils by microglial cells. *J. Alzheimers Dis.* 58 253–261. 10.3233/JAD-170108 28387679

[B2] Andre C. Tomadesso C. de Flores R. Branger P. Rehel S. Mezenge F. (2019). Brain and cognitive correlates of sleep fragmentation in elderly subjects with and without cognitive deficits. *Alzheimers Dement. (Amst.)* 11 142–150. 10.1016/j.dadm.2018.12.009 30788411 PMC6369144

[B3] Ba L. Huang L. He Z. Deng S. Xie Y. Zhang M. (2021). Does chronic sleep fragmentation lead to Alzheimer’s disease in young wild-type mice? *Front. Aging Neurosci.* 13:759983. 10.3389/fnagi.2021.759983 34992526 PMC8724697

[B4] Bero A. W. Yan P. Roh J. H. Cirrito J. R. Stewart F. R. Raichle M. E. (2011). Neuronal activity regulates the regional vulnerability to amyloid-beta deposition. *Nat. Neurosci.* 14 750–756. 10.1038/nn.2801 21532579 PMC3102784

[B5] Carter M. E. Brill J. Bonnavion P. Huguenard J. R. Huerta R. de Lecea L. (2012). Mechanism for hypocretin-mediated sleep-to-wake transitions. *Proc. Natl. Acad. Sci. U.S.A.* 109 E2635–E2644. 10.1073/pnas.1202526109 22955882 PMC3465396

[B6] Chemelli R. M. Willie J. T. Sinton C. M. Elmquist J. K. Scammell T. Lee C. (1999). Narcolepsy in orexin knockout mice: Molecular genetics of sleep regulation. *Cell* 98 437–451. 10.1016/S0092-8674(00)81973-X 10481909

[B7] Chou T. C. Lee C. E. Lu J. Elmquist J. K. Hara J. Willie J. T. (2001). Orexin (hypocretin) neurons contain dynorphin. *J. Neurosci.* 21:RC168. 10.1523/JNEUROSCI.21-19-j0003.2001 11567079 PMC6762880

[B8] Deurveilher S. Antonchuk M. Saumure B. S. C. Baldin A. Semba K. (2021). No loss of orexin/hypocretin, melanin-concentrating hormone or locus coeruleus noradrenergic neurons in a rat model of chronic sleep restriction. *Eur. J. Neurosci.* 54 6027–6043. 10.1111/ejn.15412 34355453

[B9] Duncan M. J. Guerriero L. E. Kohler K. Beechem L. E. Gillis B. D. Salisbury F. (2022). Chronic fragmentation of the daily sleep-wake rhythm increases amyloid-beta levels and neuroinflammation in the 3xTg-AD mouse model of Alzheimer’s disease. *Neuroscience* 481 111–122. 10.1016/j.neuroscience.2021.11.042 34856352 PMC8941625

[B10] Eggermann E. Serafin M. Bayer L. Machard D. Saint-Mleux B. Jones B. E. (2001). Orexins/hypocretins excite basal forebrain cholinergic neurones. *Neuroscience* 108 177–181. 10.1016/S0306-4522(01)00512-7 11734353

[B11] Fan Y. Jiang E. Gao H. Bigalke J. Chen B. Yu C. (2021). Activation of orexin system stimulates CaMKII expression. *Front. Physiol.* 12:698185. 10.3389/fphys.2021.698185 34276418 PMC8282234

[B12] Gabelle A. Jaussent I. Bouallegue F. B. Lehmann S. Lopez R. Barateau L. (2019). Reduced brain amyloid burden in elderly patients with narcolepsy type 1. *Ann. Neurol.* 85 74–83. 10.1002/ana.25373 30387527

[B13] Geula C. Mesulam M. M. Saroff D. M. Wu C. K. (1998). Relationship between plaques, tangles, and loss of cortical cholinergic fibers in Alzheimer disease. *J. Neuropathol. Exp. Neurol.* 57 63–75. 10.1097/00005072-199801000-00008 9600198

[B14] Gil-Bea F. J. Gerenu G. Aisa B. Kirazov L. P. Schliebs R. Ramirez M. J. (2012). Cholinergic denervation exacerbates amyloid pathology and induces hippocampal atrophy in Tg2576 mice. *Neurobiol. Dis.* 48 439–446. 10.1016/j.nbd.2012.06.020 22759926

[B15] Hampel H. Mesulam M. M. Cuello A. C. Farlow M. R. Giacobini E. Grossberg G. T. (2018). The cholinergic system in the pathophysiology and treatment of Alzheimer’s disease. *Brain* 141 1917–1933. 10.1093/brain/awy132 29850777 PMC6022632

[B16] Hara J. Beuckmann C. T. Nambu T. Willie J. T. Chemelli R. M. Sinton C. M. (2001). Genetic ablation of orexin neurons in mice results in narcolepsy, hypophagia, and obesity. *Neuron* 30 345–354. 10.1016/S0896-6273(01)00293-8 11394998

[B17] Hartig W. Saul A. Kacza J. Grosche J. Goldhammer S. Michalski D. (2014). Immunolesion-induced loss of cholinergic projection neurones promotes beta-amyloidosis and tau hyperphosphorylation in the hippocampus of triple-transgenic mice. *Neuropathol. Appl. Neurobiol.* 40 106–120. 10.1111/nan.12050 23566195

[B18] Kang J. E. Lim M. M. Bateman R. J. Lee J. J. Smyth L. P. Cirrito J. R. (2009). Amyloid-beta dynamics are regulated by orexin and the sleep-wake cycle. *Science* 326 1005–1007. 10.1126/science.1180962 19779148 PMC2789838

[B19] Laursen B. Mork A. Plath N. Kristiansen U. Bastlund J. F. (2013). Cholinergic degeneration is associated with increased plaque deposition and cognitive impairment in APPswe/PS1dE9 mice. *Behav. Brain Res.* 240 146–152. 10.1016/j.bbr.2012.11.012 23178660

[B20] Lee C. Y. Landreth G. E. (2010). The role of microglia in amyloid clearance from the AD brain. *J. Neural Transm. (Vienna)* 117 949–960. 10.1007/s00702-010-0433-4 20552234 PMC3653296

[B21] Li M. Meng Y. Chu B. Shen Y. Xue X. Song C. (2020). Orexin-A exacerbates Alzheimer’s disease by inducing mitochondrial impairment. *Neurosci. Lett.* 718:134741. 10.1016/j.neulet.2020.134741 31927055

[B22] Li Y. Panossian L. A. Zhang J. Zhu Y. Zhan G. Chou Y. T. (2014). Effects of chronic sleep fragmentation on wake-active neurons and the hypercapnic arousal response. *Sleep* 37 51–64. 10.5665/sleep.3306 24470695 PMC3902866

[B23] Lim A. S. Kowgier M. Yu L. Buchman A. S. Bennett D. A. (2013). Sleep fragmentation and the risk of incident Alzheimer’s disease and cognitive decline in older persons. *Sleep* 36 1027–1032. 10.5665/sleep.2802 23814339 PMC3669060

[B24] Lin L. Faraco J. Li R. Kadotani H. Rogers W. Lin X. (1999). The sleep disorder canine narcolepsy is caused by a mutation in the hypocretin (orexin) receptor 2 gene. *Cell* 98 365–376. 10.1016/S0092-8674(00)81965-0 10458611

[B25] Ma Z. Jiang W. Zhang E. E. (2016). Orexin signaling regulates both the hippocampal clock and the circadian oscillation of Alzheimer’s disease-risk genes. *Sci. Rep.* 6:36035. 10.1038/srep36035 27796320 PMC5086843

[B26] Mesulam M. Shaw P. Mash D. Weintraub S. (2004). Cholinergic nucleus basalis tauopathy emerges early in the aging-MCI-AD continuum. *Ann. Neurol.* 55 815–828. 10.1002/ana.20100 15174015

[B27] Minakawa E. N. Miyazaki K. Maruo K. Yagihara H. Fujita H. Wada K. (2017). Chronic sleep fragmentation exacerbates amyloid beta deposition in Alzheimer’s disease model mice. *Neurosci. Lett.* 653 362–369. 10.1016/j.neulet.2017.05.054 28554860

[B28] Nishino S. (2007). Clinical and neurobiological aspects of narcolepsy. *Sleep Med.* 8 373–399. 10.1016/j.sleep.2007.03.008 17470414 PMC1978248

[B29] Oh J. Eser R. A. Ehrenberg A. J. Morales D. Petersen C. Kudlacek J. (2019). Profound degeneration of wake-promoting neurons in Alzheimer’s disease. *Alzheimers Dement.* 15 1253–1263. 10.1016/j.jalz.2019.06.3916 31416793 PMC6801040

[B30] Oka M. Fujisaki N. Maruko-Otake A. Ohtake Y. Shimizu S. Saito T. (2017). Ca2+/calmodulin-dependent protein kinase II promotes neurodegeneration caused by tau phosphorylated at ser262/356 in a transgenic *Drosophila* model of tauopathy. *J. Biochem.* 162 335–342. 10.1093/jb/mvx038 28992057 PMC5892399

[B31] Opazo P. Viana da Silva S. Carta M. Breillat C. Coultrap SJ. Grillo-Bosch D. (2018). CaMKII metaplasticity drives abeta oligomer-mediated synaptotoxicity. *Cell Rep.* 23 3137–3145. 10.1016/j.celrep.2018.05.036 29898386 PMC6089247

[B32] Ovsepian S. V. Antyborzec I. O’Leary V. B. Zaborszky L. Herms J. Oliver Dolly J. (2014). Neurotrophin receptor p75 mediates the uptake of the amyloid beta (Abeta) peptide, guiding it to lysosomes for degradation in basal forebrain cholinergic neurons. *Brain Struct. Funct.* 219 1527–1541. 10.1007/s00429-013-0583-x 23716278 PMC4675333

[B33] Owen J. E. Zhu Y. Fenik P. Zhan G. Bell P. Liu C. (2021). Late-in-life neurodegeneration after chronic sleep loss in young adult mice. *Sleep* 44:zsab057. 10.1093/sleep/zsab057 33768250 PMC8361366

[B34] Panossian L. Fenik P. Zhu Y. Zhan G. McBurney M. W. Veasey S. (2011). SIRT1 regulation of wakefulness and senescence-like phenotype in wake neurons. *J. Neurosci.* 31 4025–4036. 10.1523/JNEUROSCI.5166-10.2011 21411645 PMC3065120

[B35] Peyron C. Tighe D. K. van den Pol A. N. de Lecea L. Heller H. C. Sutcliffe J. G. (1998). Neurons containing hypocretin (orexin) project to multiple neuronal systems. *J. Neurosci.* 18 9996–10015. 10.1523/JNEUROSCI.18-23-09996.1998 9822755 PMC6793310

[B36] Plascencia-Villa G. Perry G. (2021). Preventive and therapeutic strategies in Alzheimer’s disease: Focus on oxidative stress, redox metals, and ferroptosis. *Antioxid. Redox Signal.* 34 591–610. 10.1089/ars.2020.8134 32486897 PMC8098758

[B37] Qiu H. Zhong R. Liu H. Zhang F. Li S. Le W. (2016). Chronic sleep deprivation exacerbates learning-memory disability and Alzheimer’s disease-like pathologies in abetaPP(swe)/PS1(DeltaE9) mice. *J. Alzheimers Dis.* 50 669–685. 10.3233/JAD-150774 26757041

[B38] Ramos-Rodriguez J. J. Pacheco-Herrero M. Thyssen D. Murillo-Carretero M. I. Berrocoso E. Spires-Jones T. L. (2013). Rapid beta-amyloid deposition and cognitive impairment after cholinergic denervation in APP/PS1 mice. *J. Neuropathol. Exp. Neurol.* 72 272–285. 10.1097/NEN.0b013e318288a8dd 23481704 PMC3612835

[B39] Roh J. H. Huang Y. Bero A. W. Kasten T. Stewart F. R. Bateman R. J. (2012). Disruption of the sleep-wake cycle and diurnal fluctuation of beta-amyloid in mice with Alzheimer’s disease pathology. *Sci. Transl. Med.* 4:150ra22. 10.1126/scitranslmed.3004291 22956200 PMC3654377

[B40] Roh J. H. Jiang H. Finn M. B. Stewart F. R. Mahan T. E. Cirrito J. R. (2014). Potential role of orexin and sleep modulation in the pathogenesis of Alzheimer’s disease. *J. Exp. Med.* 211 2487–2496. 10.1084/jem.20141788 25422493 PMC4267230

[B41] Scammell T. E. Matheson J. K. Honda M. Thannickal T. C. Siegel J. M. (2012). Coexistence of narcolepsy and Alzheimer’s disease. *Neurobiol. Aging* 33 1318–1319. 10.1016/j.neurobiolaging.2010.12.008 21257235 PMC8720268

[B42] Shaffery J. P. Allard J. S. Manaye K. F. Roffwarg H. P. (2012). Selective rapid eye movement sleep deprivation affects cell size and number in kitten locus coeruleus. *Front. Neurol.* 3:69. 10.3389/fneur.2012.00069 22615706 PMC3351802

[B43] Sinton C. M. Kovakkattu D. Friese R. S. (2009). Validation of a novel method to interrupt sleep in the mouse. *J. Neurosci. Methods* 184 71–78. 10.1016/j.jneumeth.2009.07.026 19646474

[B44] Sportiche N. Suntsova N. Methippara M. Bashir T. Mitrani B. Szymusiak R. (2010). Sustained sleep fragmentation results in delayed changes in hippocampal-dependent cognitive function associated with reduced dentate gyrus neurogenesis. *Neuroscience* 170 247–258. 10.1016/j.neuroscience.2010.06.038 20600652 PMC2926207

[B45] Stanley E. M. Fadel J. (2012). Aging-related deficits in orexin/hypocretin modulation of the septohippocampal cholinergic system. *Synapse* 66 445–452. 10.1002/syn.21533 22213437 PMC3292656

[B46] Stokin G. B. Lillo C. Falzone T. L. Brusch R. G. Rockenstein E. Mount S. L. (2005). Axonopathy and transport deficits early in the pathogenesis of Alzheimer’s disease. *Science* 307 1282–1288. 10.1126/science.1105681 15731448

[B47] Takahashi K. Lin J. S. Sakai K. (2008). Neuronal activity of orexin and non-orexin waking-active neurons during wake-sleep states in the mouse. *Neuroscience* 153 860–870. 10.1016/j.neuroscience.2008.02.058 18424001

[B48] Torrealba F. Yanagisawa M. Saper C. B. (2003). Colocalization of orexin a and glutamate immunoreactivity in axon terminals in the tuberomammillary nucleus in rats. *Neuroscience* 119 1033–1044. 10.1016/S0306-4522(03)00238-0 12831862

[B49] Turnbull M. T. Boskovic Z. Coulson E. J. (2018). Acute down-regulation of BDNF signaling does not replicate exacerbated amyloid-beta levels and cognitive impairment induced by cholinergic basal forebrain lesion. *Front. Mol. Neurosci.* 11:51. 10.3389/fnmol.2018.00051 29520217 PMC5827359

[B50] West M. J. Gundersen H. J. (1990). Unbiased stereological estimation of the number of neurons in the human *Hippocampus*. *J. Comp. Neurol.* 296 1–22. 10.1002/cne.902960102 2358525

[B51] Wu M. Zaborszky L. Hajszan T. van den Pol A. N. Alreja M. (2004). Hypocretin/orexin innervation and excitation of identified septohippocampal cholinergic neurons. *J. Neurosci.* 24 3527–3536. 10.1523/JNEUROSCI.5364-03.2004 15071100 PMC6729747

[B52] Wurtman R. J. (2006). Narcolepsy and the hypocretins. *Metabolism* 55(10 Suppl. 2) S36–S39. 10.1016/j.metabol.2006.07.011 16979425

[B53] Xie Y. Ba L. Wang M. Deng S. Y. Chen S. M. Huang L. F. (2020). Chronic sleep fragmentation shares similar pathogenesis with neurodegenerative diseases: Endosome-autophagosome-lysosome pathway dysfunction and microglia-mediated neuroinflammation. *CNS Neurosci. Ther.* 26 215–227. 10.1111/cns.13218 31549780 PMC6978272

[B54] Zhang J. Zhu Y. Zhan G. Fenik P. Panossian L. Wang M. M. (2014). Extended wakefulness: Compromised metabolics in and degeneration of locus ceruleus neurons. *J. Neurosci.* 34 4418–4431. 10.1523/JNEUROSCI.5025-12.2014 24647961 PMC3960479

[B55] Zhao H. Y. Wu H. J. He J. L. Zhuang J. H. Liu Z. Y. Huang L. Q. (2017). Chronic sleep restriction induces cognitive deficits and cortical beta-amyloid deposition in mice via BACE1-antisense activation. *CNS Neurosci. Ther.* 23 233–240. 10.1111/cns.12667 28145081 PMC6492718

[B56] Zhu Y. Fenik P. Zhan G. Somach R. Xin R. Veasey S. (2016). Intermittent short sleep results in lasting sleep wake disturbances and degeneration of locus coeruleus and orexinergic neurons. *Sleep* 39 1601–1611. 10.5665/sleep.6030 27306266 PMC4945320

[B57] Zhu Y. Fenik P. Zhan G. Xin R. Veasey S. C. (2015). Degeneration in arousal neurons in chronic sleep disruption modeling sleep apnea. *Front. Neurol.* 6:109. 10.3389/fneur.2015.00109 26074865 PMC4443725

[B58] Zhu Z. Xu L. Cao D. Song C. Wang Y. Li M. (2021). Effect of orexin-A on mitochondrial biogenesis, mitophagy and structure in HEK293-APPSWE cell model of Alzheimer’s disease. *Clin. Exp. Pharmacol. Physiol.* 48 355–360. 10.1111/1440-1681.13424 33080054

